# Clear lens extraction and intraocular lens implantation in a case of microspherophakia with secondary angle closure glaucoma

**DOI:** 10.4103/0301-4738.58477

**Published:** 2010

**Authors:** Harsha Bhattacharjee, Kasturi Bhattacharjee, Jnanankar Medhi, Sushobhan DasGupta

**Affiliations:** Sri Sankaradeva Nethralaya, Guwahati, Assam, India

**Keywords:** Capsulorhexis, clear lens extraction, spherophakia

## Abstract

Phacoemulsification with implantation of single-piece acrylic foldable intraocular lens (IOL) in a 19-year-old boy with microspherophakia, high myopia and angle closure glaucoma is described. The associated myopia and angle closure glaucoma was severely compromising the quality of life. Post-surgical visual recovery was 20/20 with sustained normal intraocular pressure. Management of such cases at times calls for innovations in current surgical technique.

Microspherophakia is an uncommon bilateral congenital abnormality of the crystalline lens. Defective development of the zonules results in their deficiency, increased length, weakness and non-attachment of posterior zonules to the ciliary processes. This leads to formation of a small spherical lens which is devoid of any cortico-nuclear demarcation. The condition may be isolated, idiopathic and familial anomaly or it may be associated with systemic defects like Marfan's syndrome, Weil-Marchesani syndrome, hyperlysinemia and congenital rubella. Microspherophakia results in lenticular myopia and late development of lens dislocation, usually inferiorly. Phakic pupillary block glaucoma and high lenticular myopia are common complications.[1–5] Management of such cases at times calls for innovations in current surgical techniques as reported in this case report.

## Case Report

**Figure 1:**
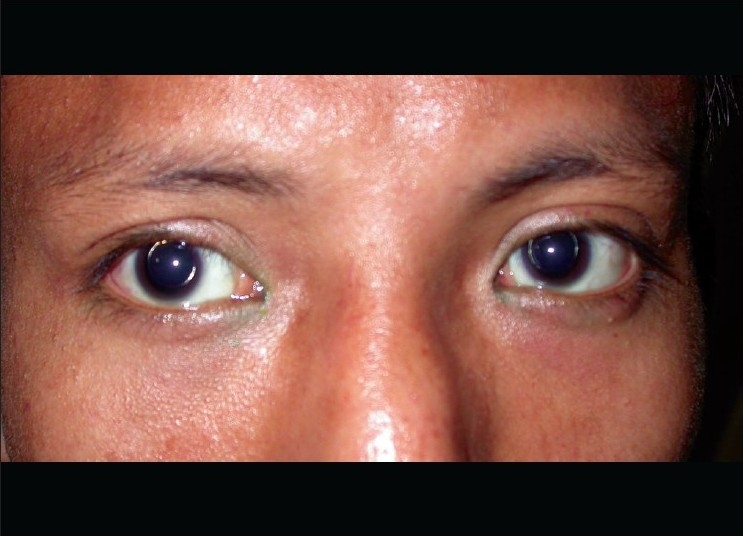
External photograph of a case of bilateral microspherophakia. Golden reflex seen in the lens margin following mydriasis

**Figure 2:**
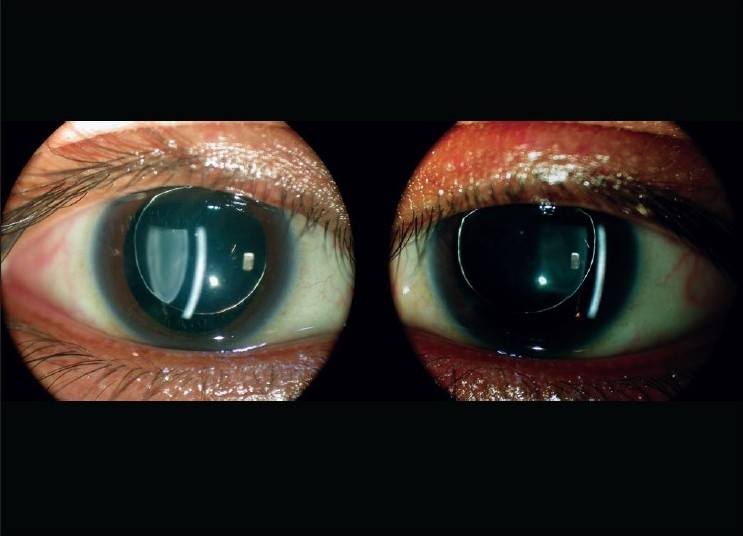
Slit-lamp optical section showing small thick spherical lens with stretched zonules (right eye)

**Figure 3:**
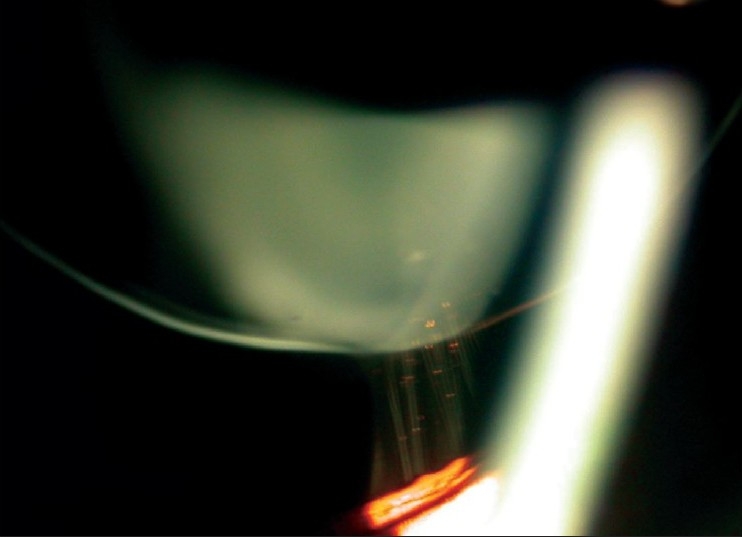
Slit-lamp photograph showing scanty, long zonules (right eye). Some zonules having isolated pigment clumps

**Figure 4:**
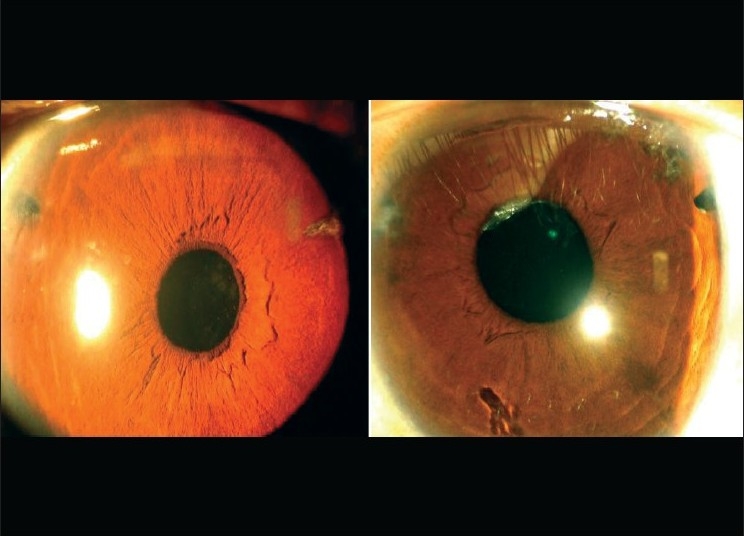
Slit-lamp photo showing both eyes at one-year follow-up

A 19-year-old boy reported to our institution with complaints of poor vision in both eyes since early childhood. He had a history of recurrent attacks of blurring of vision, associated with headache, irritation and redness of eyes. On examination, the best-corrected visual acuity (BCVA) with contact lens was 20/60, N12 in right eye (RE) and 20/80, N18 in left eye (LE). Refractive error was -23.00/-1.00 × 80° (RE) and -24.00/-1.00 ×100°(LE). He achieved N8 in near reading by bringing matter close to face. On slit-lamp examination (SLE), the anterior chamber (AC) was shallow in both eyes. Intraocular pressure (IOP) with applanation tonometer (AT) was 30 and 34 mm Hg in RE and LE respectively. Gonioscopy detected convex iris configuration and Grade II Shaffer's angle with tilted mirror view. Ultrasound biomicroscopy (UBM) analysis detected small spherical lens with a lens thickness of 6 mm (RE) and 5.9 mm (LE) and almost 360° narrow angle. AC depth was 2.00 mm in the RE and 2.04 mm in the LE. Autokeratometry value of corneal curvature was 44.50D/45.00D (vertical/horizontal) and 44.00D/45.50D (vertical/horizontal) in the RE and LE respectively. Axial length of the eye was determined by laser-interferometry and the values were 24.54 mm (RE), and 24.84 mm (LE). Systemic evaluation did not detect any abnormality. The case was diagnosed as pupillary block glaucoma, microspherophakia and lenticular myopia (both eyes). Nd:YAG-laser peripheral iridotomy (PI) was done in both eyes. On the third day after the procedure, the IOP reduced to 10 mm Hg (AT) in both eyes. Subsequently, following mydriasis, the entire lens equator was visualized under the slit lamp [Fig. 1] and both the lenses were found to have minimal nasal subluxation [Figs. 2–4]. The indirect ophthalmoscopic examination of the posterior segment was unremarkable with the cup-disc ratio being within normal limits in both eyes. Emmetropic intraocular lens (IOL) power calculation using SRK-II formula was +16.50 in the RE and + 15.50 in the LE (A-constant 118.4). Potential acuitymetry showed a visual potential of 20/20 in each eye.

Considering compromised quality of life with reference to dependence on contact lenses or glasses, lack of confidence in independent living, and good visual potential in eyes, clear lens extraction and IOL implantation was suggested in both eyes and accordingly the LE was operated first, followed by the other.

### Surgical Procedure

**Figure 5:**
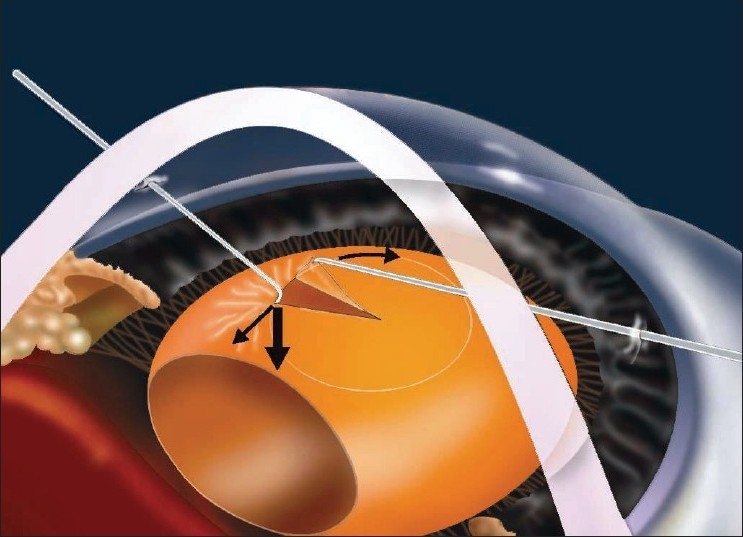
Diagram showing mechanics of capsulorrhexis in microspherophakia

The patient underwent difficult, uneventful phacoaspiration with IOL implantation under topical anesthesia (HB), through a 2.75-mm superior clear corneal tunnel and two corneal side ports. Puncturing of the capsule and initiation of capsulorrhexis was difficult, as the anterior capsule was less elastic and relatively unsupported and cystitome pressure made an umbilicated depression in the anterior capsule with radiating folds. The lens was unstable and bimanual capsulorrhexis was done by supporting the capsulorhexis margin with an iris hook through the side port [Fig. 5]. A capsulorhexis of size 4.5 mm (approximately) was made. After hydroprocedure and phacoaspiration, a foldable IOL of + 15.50D (AcrySof^®^ SA60AT Alcon Lab. Fortworth. Tx, USA) was implanted directly into the bag. Tight wound security and deep AC were maintained critically during the procedure. The capsular bag was small, but somehow accommodated the intraocular lens. On the first postoperative day, AC was found to be deep, quiet and with presence of iridodonesis. The IOL was well centered. The BCVA (Plano) at two weeks was 20/20.

The same procedure was repeated in the other eye with similar capsulorrhexis technique and fashioning a capsulorhexis of approximately 4.5 mm. An IOL of +16.50D (AcrySof^®^ SA60AT) was implanted and a BCVA (-0.75 cylinder in 20°) of 20/20 was achieved at two weeks.

**Figure 6:**
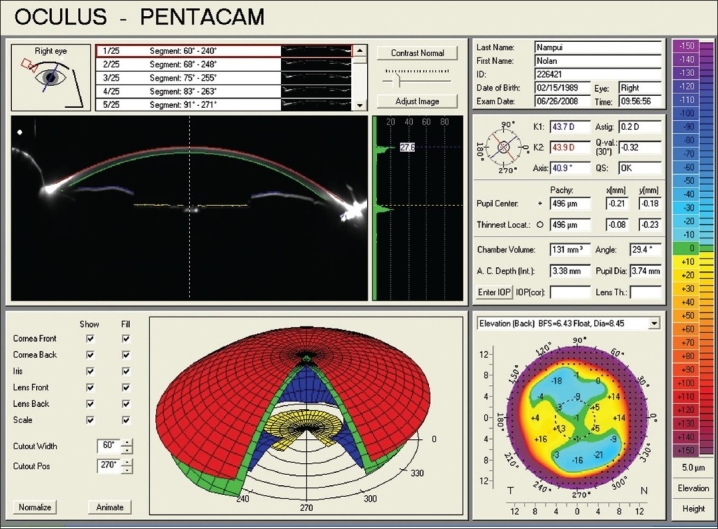
Scheimpflug imaging of the right eye, showing satisfactory anterior chamber depth and angle configuration

At the one-year follow-up, BCVA was maintained at 20/20 in both eyes. SLE revealed quiet eyes with patent PI [Fig. 4]. There was increase of AC depth to 3.38 mm (RE) and 3.65 mm (LE) and angle configuration was 29.4° (RE) and 40.2° (LE) with Scheimpflug camera photograph [Fig. 6].

## Discussion

Faulty development of lens zonules during embryogenesis is believed to be the cause of microspherophakia which can cause pupillary block glaucoma. Miotics aggravate the condition by increasing the pupillary block and allowing further forward displacement of the crystalline lens. Cycloplegics are the treatment of choice. However, laser-iridotomy, trabeculectomy,[6] were found useful in relieving angle closure glaucoma in such patients. Management of associated high myopia is always a challenge. Clear lens extraction through anterior route, pars plana lensectomy with or without IOL implantation are the treatment options for myopia and glaucoma in microspherophakia.[7–9] Indications for lens extraction in such cases are cataract, corneo-lenticular touch, high myopia, intermittent pupillary block and secondary glaucoma. Literature search revealed only few case reports, where clear lens phacoemulsification was done with appropriate IOL implantation, with excellent postoperative visual recovery.[10,11] In the present case, pupillary block glaucoma was successfully treated with Nd:YAG-Laser PI followed by refractive error correction through clear lens extraction with IOL implantation. During the surgery, the capsulorrhexis needed an innovation in the form of using with an iris hook [Fig. 5] to stabilize the lens. The option of stabilizing the capsular bag with capsular tension ring (CTR) could not be explored by us, because of nonavailability of such a small CTR[7] and also with apprehension that it will further reduce the capsular bag volume. The acrylic foldable lens was well accommodated in the small capsular bag with the unfolded haptics being in touch with the edge of the optics. The IOL was well centered in spite of zonular instability, but clinically phacodonesis persisted in the postoperative period, without compromising the vision. A similar procedure has been described by Khokhar *et al*.[10] but in their case there was residual myopia and underestimated visual potential acuitymetry result. In the present case there was no residual myopia, suggesting that the IOL power calculation by the optical biometer using SRK-II formula as appropriate.

We believe Nd:YAG PI, clear lens extraction and IOL implantation may be a valid treatment option for treating pupillary block glaucoma and high myopia in microspherophakia.
